# Pancreatic cancer adjuvant radiotherapy target volume design: based on the postoperative local recurrence spatial location

**DOI:** 10.1186/s13014-016-0714-7

**Published:** 2016-10-19

**Authors:** Wei Yu, Wei Hu, Yongjie Shui, Xiaoyang Zhu, Chao Li, Xiaoqiu Ren, Xueli Bai, Risheng Yu, Li Shen, Tingbo Liang, Lei Zheng, Qichun Wei

**Affiliations:** 1 Department of Radiation Oncology, the Second Affiliated Hospital, Zhejiang University School of Medicine, Hangzhou, Zhejiang 310009 People’s Republic of China; 2 Department of Hepatobiliary and Pancreatic Surgery, the Second Affiliated Hospital, Zhejiang University School of Medicine, Hangzhou, Zhejiang 310009 People’s Republic of China; 3 Department of Radiology, the Second Affiliated Hospital, Zhejiang University School of Medicine, Hangzhou, Zhejiang 310009 People’s Republic of China; 4 The Sidney Kimmel Comprehensive Cancer Center, the Johns Hopkins University School of Medicine, Baltimore, MD USA; 5 Department of Radiation Oncology, the Second Affiliated Hospital, Ministry of Education Key Laboratory of Cancer Prevention and Intervention, Zhejiang University School of Medicine, Jiefang Road 88, Hangzhou, 310009 People’s Republic of China

**Keywords:** Pancreatic cancer, Local recurrence pattern, Spatial location, Target volume, Adjuvant radiotherapy

## Abstract

**Objectives:**

To explore the areas at highest risk for postoperative pancreatic cancer local recurrence according to the spatial location of local failures, with the aim to provide a precise target volume for pancreatic cancer adjuvant radiotherapy.

**Methods:**

Patients with pancreatic cancer who had undergone surgery for the primary tumor in pancreas at our institution from January 2010 to August 2015 were retrospectively analyzed. All local recurrences were plotted on the computed tomography (CT) image of a representative patient according to their relative coordinates to superior mesenteric artery (SMA) or celiac axis (CA). Adjuvant radiation clinical target volume (CTV)-90 and CTV-80 were created to cover 90 % and 80 % plotted recurrences. This planning approach was applied in four simulated cases with comparison to the plan according to RTOG 0848 contouring consensus guidelines. Raystation v4.5.1.14 was used for analyzing high throughput physics data.

**Results:**

Eighty-three patients with local recurrence were included from 305 postoperative pancreatic cancer patients who did not receive adjuvant radiotherapy. Thirty-one (37 %) patients did not have adjuvant therapy at all, 52 (63 %) patients undergone adjuvant chemotherapy alone. Spatial location of local failure was created. Most recurrences occurred near CA or SMA. CTV-90 was generated through expanding the combined SMA and CA contours by 30 mm right-lateral, 21 mm left-lateral, 20 mm anterior, 13 mm posterior, 10 mm superior, and 20 mm inferior. CTV-80, smaller in volume, was also created for simultaneous integrated boost. Through comparison and analysis of the simulated cases, the radiation volumes proposed were much smaller than those with RTOG 0848 contouring consensus guidelines (average volume: PTV-80 = 120 ml, PTV-90 = 220 ml, RTOG PTV = 490 ml). Accordingly, the organs at risk received less irradiation dose with the proposed CTV-90 and CTV-80.

**Conclusions:**

Smaller adjuvant radiotherapy CTVs targeting the high-risk local failure areas of postoperative pancreatic cancer were proposed, according to the three-dimensional spatial location of local recurrences. This may help to minimize radiation-related toxicities, achieve dose escalation, and finally reduce local recurrence.

## Introduction

Pancreatic cancer (PCA) is the fourth deadliest solid malignancies in the United States. It is estimated that 48960 new cases will be diagnosed as PCA in the USA in 2015. Among them, 40560 will die from this disease [1]. In China, PCA is the 9th most commonly diagnosed cancer and the sixth leading cause of cancer death. The National Central Cancer Registry (NCCR) of China predicted that there will be approximately 90100 newly diagnosed pancreatic cancer cases and 79400 cases will die from this disease in 2015 [2]. Resectability of the cancer is important to stratify survival. However, at the time of diagnosis, only 20 % of patients are able to undergo surgical resection. Even in these patients, the 5 years overall survival rate is only 10–20 %. Although most of patients died from distant metastases, it has been verified that the incidence of local recurrence were up to 20 % to 60 % [3–5], and autopsy studies reported even higher rates of local recurrence [6]. These findings have highlighted the importance of local control in resectable pancreatic cancer.

The standard options of adjuvant therapy for pancreatic cancer include chemotherapy and chemoradiation (CRT). However, the role of radiation therapy in the adjuvant therapy is still controversial [7–12]. One of the explanations is the insufficient of radiation dose in conventional radiotherapy, due to the normal structure dose constraints. Furthermore, the integration of approximately 6 weeks of chemoradiation (CRT) (45 Gy directed to the tumor bed, surgical anastomoses and peripancreatic nodes with boost of 5 to 15 Gy to the tumor bed) has high toxicity which decreases quality of life and delays the delivery of full dose chemotherapy [7].

Nevertheless, technological developments in image guidance and motion management have enabled the precise radiotherapy. So smaller target volume is possible in order to minimize treatment-related toxicity. Stereotactic body radiotherapy (SBRT) represents a novel field of radiation therapy and is a tumor-ablative radiation modality employing multiple non-coplanar fixed beams or arc fields to damage the target accurately and precisely with a high dose while geometrically sparing adjacent normal tissues. It enables accomplishing radiotherapy in a week without interrupt of the chemotherapy course. However, the standard dose and volume for SBRT in pancreatic cancer are to be explored.

To rule out the impact of radiotherapy on the site of recurrence, only those patients who did not receive adjuvant radiotherapy were included in this study. We map the postoperative local recurrences of pancreatic cancer patients with respect to major arteries in radiographic imaging to explore the areas at the highest risk for local recurrence, and then to provide a suitable target volume for adjuvant radiotherapy of pancreatic cancer, and to achieve dose escalation. This may allow decreased treatment-related toxicity and increased probability of disease control.

## Methods and materials

### Patients

This study was approved by the Institutional Review Board of the Second Affiliated Hospital, Zhejiang University School of Medicine (SAHZU). From January 2010 to August 2015, 305 patients had undergone surgery for the primary pancreatic cancer at SAHZU, histopathology diagnosis were achieved after surgery. The medical records were retrospectively reviewed. Patients were included if they met the following inclusion criteria: 1) patients after surgery for the primary tumor in pancreas; 2) patients with local recurrence, with or without distant metastasis; 3) patients did not receive neoadjuvant or adjuvant radiation. On the other hand, patients were excluded based on the following: 1) patients after Palliative surgery; 2) patients had no follow-up abdominal Computed Tomography (CT) or Magnetic Resonance Imaging (MRI) in our institution; 3) cases without local recurrence. Local recurrence was defined as progression of soft tissue at the resection area or surrounding the peripancreatic vessels and progression of retroperitoneal lymph nodes in follow-up imaging. A radiologist specializing in abdominal neoplasms imaging identified the recurrences. Rising of CA199 and deterioration of patients’ symptom and physical condition were also helpful. Multidisciplinairy team was attended for uncertain case. Patients were classified into three groups: pancreatic head cancer (PHC), pancreatic body cancer (PBC) and pancreatic tail cancer (PTC). In each group, patients were categorized into subgroups based on adjuvant therapy they received: adjuvant chemotherapy alone (CTA) or no adjuvant therapy (NA). The following data were collected for each local recurrent patient: age, sex, tumor diameter, T stage, N stage, resection margins (R0: grossly complete resection with microscopically negative margins; R1: tumor invasion within 1 mm from the resection margins; R2: grossly incomplete resection), histological type, comprehensive treatment pattern, type of recurrence and resection type.

### Three-dimensional local recurrence map

All local recurrences were plotted on the CT image of a representative patient according to their relative coordinates to superior mesenteric artery (SMA) or celiac axis (CA) to construct a three-dimensional local recurrence map, which was produced by Raystation software (Raysearch, Stockholm, Sweden). The three-dimensional location of the center of recurrent tumor relative to the center of the SMA or CA of each patient with local failure was measured. To choose SMA or CA depended on which artery was closer to the tumor. SMA and CA were contoured from the origin of the arteries to 30 mm and 10 mm along the natural anatomy of the arteries inferiorly, respectively. The center of the tumor and the center of the arteries were located by Raystation software as mentioned above.

### Novel adjuvant field delineation

A radiation target volume for adjuvant radiotherapy was designed to the areas where local failures commonly occurred according to the three-dimensional local recurrence map. The center of all plotted recurrences was located by Raystation software which had been mentioned above. Then 90 % and 80 % of plotted recurrences closer to the center were produced respectively. A combined contour structure of the SMA and CA was expanded to cover 90 % and 80 % plotted recurrences, thus to generate clinical target volume (CTV)-90 and CTV-80. Planning target volume (PTV)-90 and PTV-80 was created through expanding CTV-90 and CTV-80 by 3 mm, respectively.

### Treatment planning

We applied the proposed plan in four simulated patients. Organs at risk (OARs) included the liver, stomach, small intestine, large bowel, spinal cord and kidneys. Doses constraints of OARs were based on a study of Herman et al. [13]. PTV-90 and PTV-80 were retracted to avoid the stomach, small intestine, and large bowel by 3 mm to generate PTV-90_modified and PTV-80_modified. For the adjuvant SBRT treatment plans, 25 Gy in 5-Gy fractions was delivered to PTV-90_modified, 33 Gy in 6.6 Gy fractions was delivered to PTV-80_modified as simultaneous integrated boost. More than 90 % of each target volume received 100 % of the prescription dose, and no more than 1 cc of PTV-80_modified received more than 120 % of the prescription dose. The plans were delivered in 5 consecutive days. They were not applied in real patients.

A standard radiation plan based on RTOG 0848 [14] was also employed in these four cases. Regions of interest (ROIs) included most proximal 10 mm of the CA and most proximal 30 mm of the SMA from the take-off from the aorta, portal vein(PV), preoperative tumor volume, pancreaticojejunostomy (PJ) and aorta from the most cephalad of CA, PJ or PV to the bottom of the second lumbar vertebra. Then CA, SMA and PV were expanded by 10 mm in all directions. PJ was expanded by 5–10 mm in all directions. The preoperative tumor volume was expanded by 5–10 mm in all directions. Aorta was expanded by 25 mm to the right, 10 mm to the left, 20–25 mm to the anterior, 2 mm to the posterior and 0 mm to the inferior. The expansion of aorta in the superior direction should up to the most superior slice of CA, PJ or PV expansion. The CTV was created by merging the above ROI expansions. Normal structures such as liver, stomach, small intestine, large bowel, left kidney, right kidney and spinal canal were contoured. Dose-volume constraints also followed RTOG 0848 guideline [14]. The CTV was edited to be adjacent to a dose limited normal organ if the noted expansions protruded into it to construct CTV_modified. The PTV_modified was created through expanding the CTV by 5 mm in all directions. PTV_modified was delivered 50.4 Gy in 1.8-Gy fractions in 28 consecutive daily fractions over 5 to 6 weeks. More than 90 % of the target volume received 100 % of the prescription dose.

### Statistical analysis

Normality test (Kolmogorov-Smirnov test) and homogeneity of variance test (Levene test) were conducted in measurement data such as age, tumor diameter and Relapse-free time interval among subgroups. In each test, P >0.1 was considered Gaussian distribution and homogeneity of variance, respectively. Two-group *t*-test was conducted among subgroups to compare the measurement data if they were Gaussian distribution and homogeneity of variance. Chi-square test and Fisher’s exact test was conducted among subgroups to compare enumeration data such as T stage, N stage, resection margin, histological type, comprehensive treatment pattern, resection type and type of recurrence. P < 0.05 was considered significant. IBM SPSS Statistics 19.0 was used for statistical analyses. Raystation v4.5.1.14 was used for analyzing high throughput physics data.

## Results

### Patient clinical characteristics

Of the 305 patients, 83 patients met the criteria were included in the study. Among them, 62 had PHC, 13 had PBC and 8 had PTC. Table 1 shows the baseline clinical characteristics. The measurement data such as age, tumor diameter and Relapse-free time interval in each subgroup were Gaussian distribution and homogeneity of variance. The mean recurrence-free time interval was 8.4 months (range, 1.1-43.2 month), specifically 8.4 month (range, 1.1-43.2 months), 6.8 months (range, 1.4-15.5 months), 11.1 months (range, 1.3-39.8 months) for PHC, PBC, PTC respectively. Forty five (54 %) patients was in stage N0 and 38 (46 %) was in stage N1. Sixty two (75 %) patients undergone pancreatoduodenectomy and twenty one (25 %) patients undergone distal pancreatectomy. Thirty-one (37 %) patients undergone NA, 52 (63 %) patients undergone CTA. Thirty-one (37 %) patients undergone local recurrence only. The majority of patients with PHC undergone pancreatoduodenectomy, while all patients in PBC and PTC undergone distal pancreatectomy (PHC vs PBC: p < 0.001, PHC vs PTC: p < 0.001). All other baseline clinical characteristics were comparable among patients with PHC, PBC and PTC.

**Table 1:** Baseline clinical characteristics

Characteristic	All(%) *n* = 83	PHC(%) *n* = 62	PBC(%) *n* = 13	PTC(%) *n* = 8	PHC vs PBC *p* value	PBC vs PTC *p* value	PHC vs PTC *p* value
Age, mean(year)	62	62	62	65	0.951	0.328	0.336
rang	36–81	36–81	50–70	47–75			
Sex							
Male	51(61)	37(60)	8(62)	6(75)			
Female	32(39)	25(40)	5(38)	2(25)	0.901	0.656	0.651
Tumor diameter, mean(mm)	34.8	34.4	34.2	39.3	0.963	0.32	0.261
range	17.5–80	17.5–80	21–55	25–65			
T stage							
T2	12(14)	7(11)	4(31)	1(13)			
T3	53(64)	41(66)	5(38)	7(88)			
T4	18(22)	14(23)	4(31)	0(0)	0.08	0.104	0.358
N stage							
N0	45(54)	31(50)	8(62)	6(75)			
N1	38(46)	31(50)	5(38)	2(25)	0.449	0.656	0.339
Resection marging							
R0	80(96)	59(95)	13(100)	8(100)			
R1	3(4)	3(5)	0(0)	0(0)	0.755		1
Histological type							
Ductal adenocarcinoma	76(92)	59(95)	11(85)	6(75)			
Intraductal papillary-mucinous tumor	2(2)	1(2)	0(0)	1(13)			
Mucoepidermoid carcinoma	3(4)	1(2)	1(8)	1(13)			
Neuroendocrine neoplasms	2(2)	1(2)	1(8)	0(0)	0.205	0.809	0.097
Comprehensive treatment pattern							
no adjuvant therapy	31(37)	24(39)	3(23)	4(50)			
adjuvant chemotherapy alone	52(63)	38(61)	10(77)	4(50)	0.453	0.346	0.818
Resection type							
pancreatoduodenectomy	53(64)	53(85)	0(0)	0(0)			
distal pancreatectomy	21(25)	0(0)	13(100)	8(100)			
pancreatoduodenectomy & total pancreatectomy	7(8)	7(11)	0(0)	0(0)			
pancreatoduodenectomy & distal pancreatectomy	2(2)	2(3)	0(0)	0(0)	<0.001		<0.001
Type of recurrence							
Local only	31(37)	25(40)	4(31)	2(25)			
local & metastasis	52(63)	37(60)	9(69)	6(75)	0.52	1	0.651
Relapse-free time interval, mean(month)	8.4	8.4	6.8	11.1	0.507	0.253	0.38
range	1.1–43.2	1.1–43.2	1.4–15.5	1.3–39.8			

*Abbreviations*: *PHC* pancreatic head cancer, *PBC* pancreatic body cancer, *PTC* pancreatic tail cancer

### Three-dimensional local recurrence map

**Fig. 1:**
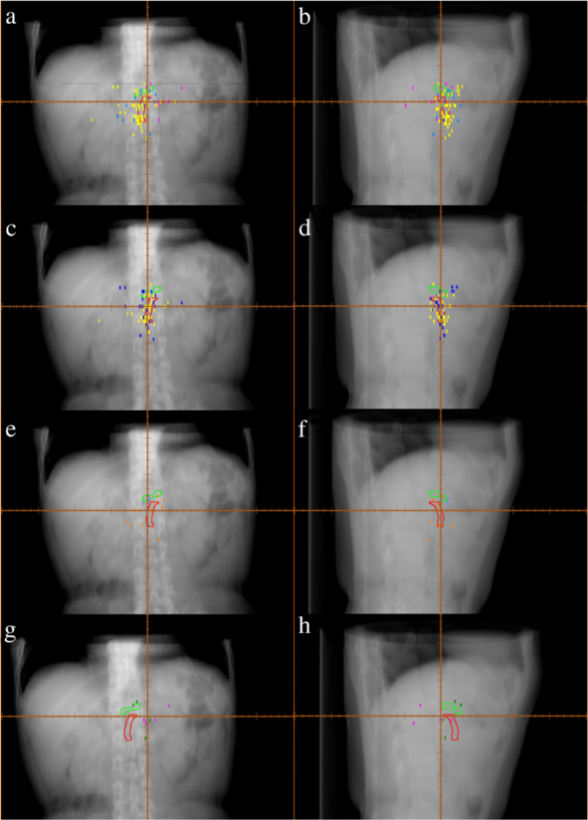
Digitally reconstructed radiograph of local recurrences. **a** Anterior-posterior and **b** lateral views of local recurrence plots with respect to the superior mesenteric artery (*red*) and celiac artery (*green*) for postoperative patients with pancreatic head(*yellow*), body(*blue*), and tail (*peach*) cancer. **c** Anterior-posterior and **d** lateral views of local recurrence plots with respect to the superior mesenteric artery (*red*) and celiac artery (*green*) for postoperative patients with chemotherapy alone (*yellow*) or no adjuvant therapy (*blue*) in pancreatic head cancer. **e** Anterior-posterior and **f** lateral views of local recurrence plots with respect to the superior mesenteric artery (*red*) and celiac artery (*green*) for postoperative patients with chemotherapy alone (*orange*) or no adjuvant therapy (*lavender*) in pancreatic head cancer. **g** Anterior-posterior and **h** lateral views of local recurrence plots with respect to the superior mesenteric artery (*red*) and celiac artery (*green*) for postoperative patients with chemotherapy alone (*peach*) or no adjuvant therapy (*dark green*) in pancreatic head cancer

Patterns of local failure by location of pancreatic cancer are shown in Fig. 1. Most recurrences occurred near CA or SMA. Sixty four patients (77 %) suffered from recurrence closer to SMA, with a mean distance from the center of SMA of 19.5 mm, 25 mm and 36.4 mm in PHC, PBC and PTC groups respectively. Nineteen patients (23 %) suffered from recurrence closer to CA, with a mean distance from the center of CA of 18.6 mm, 12.2 mm and 40.5 mm in PHC, PBC and PTC groups respectively. According to the picture and data, the recurrence pattern of PTC seems at the left-rear of SMA and CA, while the recurrence location of PHC and PBC tend to be surrounding SMA and CA.

Patterns of local failure classified by adjuvant therapy patients with PHC and PBC received are shown in Fig. 1. In patients with PHC, 24 had NA, 38 had CTA. In patients with PBC, 3 had NA, 10 had CTA. In patients with PHC, 50 (81 %) suffered from recurrence closer to SMA, with a mean distance from the center of SMA of 19.4 mm and 19.9 mm in the NA and CTA groups respectively. Twelve patients (23 %) suffered from recurrence closer to CA, with a mean distance from the center of CA of 20.6 mm and 14.4 mm in NA and CTA groups respectively. In patients with PBC, 9 (69 %) suffered from recurrence closer to SMA, with a mean distance from the center of SMA of 21.8 mm, 25.4 mm in the NA, CTA groups respectively. Four patients (31 %) suffered from recurrence closer to CA, with a mean distance from the center of CA of 12.2 mm and 12.1 mm in NA and CTA groups respectively.

Table 2 shows the comparison of baseline clinical characteristics between NA and CTA groups in pancreatic head cancer. The NA patients were older, with a median age of 65 years compared with 60 years in the CTA groups (p = 0.038). All other baseline clinical characteristics were comparable among patients receiving NA and CTA*.*

**Table 2:** Comparison of baseline clinical characteristics between NA and CTA groups in pancreatic head cancer

Characteristic	NA(%) *n* = 24	CTA(%) *n* = 38	NA vs CTA *p* value
Age, mean(year)	65	60	0.038
rang	45–81	36–74	
Sex			
Male	13(54)	24(63)	
Female	11(46)	14(37)	0.482
Tumor diameter, mean(mm)	34.1	34.5	0.884
range	20–57.5	17.5–80	
T stage			
T2	3(13)	4(11)	
T3	14(58)	27(71)	
T4	7(29)	7(18)	0.532
N stage			
N0	12(50)	19(50)	
N1	12(50)	19(50)	1
Resection marging			
R0	24(100)	35(92)	
R1	0(0)	3(8)	0.422
histological type			
Dactal adenocarcinoma	22(92)	37(97)	
Intraductal papillary-mucinous tumor	1(4)	0(0)	
Mucoepidermoid carcinoma	0(0)	1(3)	
Neuroendocrine neoplasms	1(4)	0(0)	0.331
Resection type			
pancreatoduodenectomy	19(79)	35(92)	
distal pancreatectomy	0(0)	0(0)	
pancreatoduodenectomy & total pancreatectomy	4(17)	2(5)	
pancreatoduodenectomy & distal pancreatectomy	1(4)	1(3)	0.315
Type of recurrence			
Local only	7(29)	18(47)	
local & metastasis	17(71)	20(53)	0.155
Relapse-free time interval, mean(month)	7.9	8.7	0.644
range	1.1–43.2	1.4–17.8	

*Abbreviations*: *NA* no adjuvant therapy, *CTA* adjuvant chemotherapy alone

### Production of treatment target volume

**Fig. 2:**
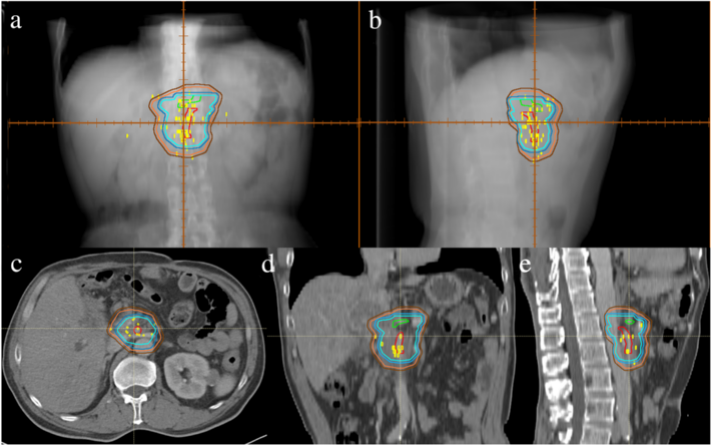
CTV-90 (*orang*e) and CTV-80 (*light blue*) which target the regions where 90 % and 80 % of recurrences occur and their corresponding PTV-90 (*brown*) and PTV-80 (*dark blue*) in one simulated patient with pancreatic head cancer in anterior-posterior (**a**), lateral (**b**), axial (**c**), coronal (**d**) and sagittal (**e**) views

By expanding the combined SMA and CA contour structure, the CTV for SBRT of pancreatic head cancer was constructed to generate a volume that included areas where failure was frequent. Ninety percent of recurrences were contained by a 30 mm right-lateral, 21 mm left-lateral, 20 mm anterior, 13 mm posterior, 10 mm superior, and 20 mm inferior expansion of the combined SMA and CA contours to generate CTV-90. Eighty percent of recurrences were contained by a 20 mm right-lateral, 18 mm left-lateral, 12 mm anterior, 12 mm posterior, 0 mm superior, and 10 mm inferior expansion of the combined SMA and CA contours to create CTV-80. PTV-90 and PTV-80 was created through expanding CTV-90 and CTV-80 by 3 mm, respectively (Fig. 2).

### Comparison of the proposed plan with standard radiation plan

**Fig. 3:**
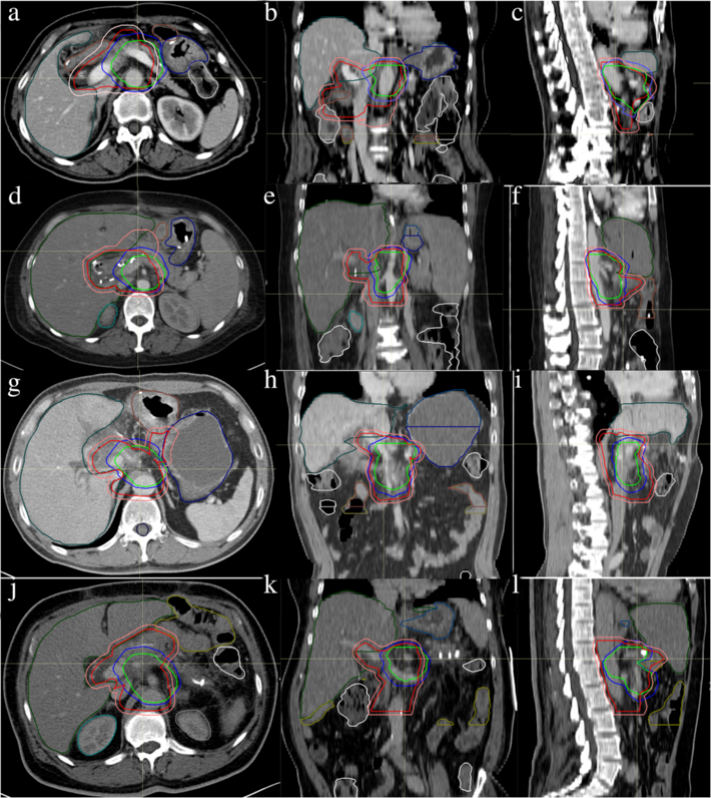
The proposed PTV-80_modified (*green*) and PTV-90_modified (*blue*) of this study are shown simultaneously with RTOG 0848 CTV_modified (red) and PTV_modified (*pink*) in 4 simulated cases. Axial, coronal and sagittal views are shown for case 1 (**a**, **b**, and **c**), case 2 (**d**, **e**, and **f**), case 3 (**g**, **h**, and **i**) and case 4 (**j**, **k** and **l**)

**Fig. 4:**
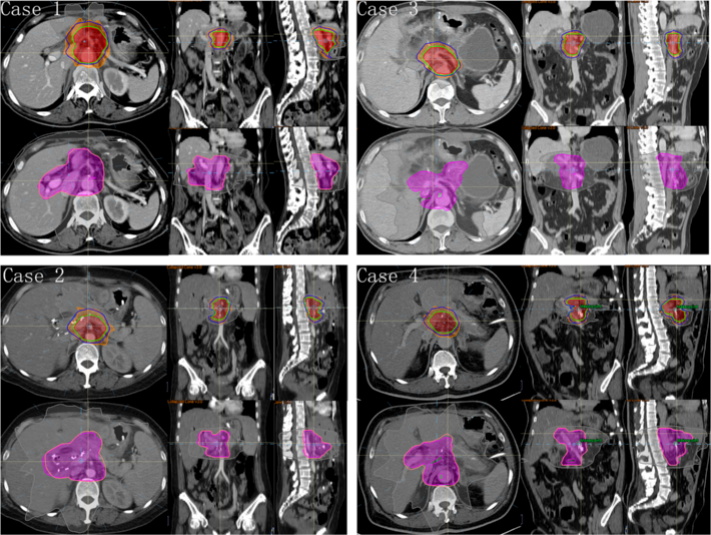
Comparison of the proposed plan (the first row in each case) and RTOG 0848 plan (the second row in each case) in 4 simulated cases. In each case, Axial, sagittal, and coronal views are shown, which are in the same section in both plans, respectively. PTV-90_modified (*blue*), PTV-80_modified (*green*), PTV_modified (*pink*), 33 Gy isodose line (*red*), 25 Gy isodose line (*orange*), 50.4 Gy isodose line (*peach*), 45.45 % isodose line (*grey*) are indicated on each plan

We applied this plan and the standard radiation plan recommended by RTOG 0848 in four simulated patients. Doses constraints of OARs of the proposed plan and RTOG 0848 are seen in Table 3. PTV-90_modified and PTV-80_modified were generated by adjusting PTV-90 and PTV-80 to avoid the stomach, jejunum, and bowel by 3 mm. For RTOG 0848 plan, CTV_modified was generated by editing CTV to be adjacent to dose-limited normal organ if it protrudes into relative structure. PTV_modified was generated through expanding CTV_modified by 5 mm (Fig. 3). The areas of the proposed PTV-90_modified and PTV-80_modifed were much smaller than RTOG 0848 PTV_modified. Figure 4 illustrates comparison of the proposed plan and RTOG 0848 plan in 4 simulated cases in the same sections. The average volume of PTV-90_modified and PTV-80_modified were 197 cc and 113 cc, respectively. However, the average volume of PTV_modified according to RTOG 0848 was 466 cc, which was much larger than the target volume in the proposed plan (Table 4). OARs and target volume in all plans reached the dosimetric constraints (Tables 3 and 5). Table 6 compares the dosimetric parameters between the proposed plan and the plan according to RTOG 0848 in the 4 simulated cases. The dose OARs received in the proposed plan was much lower than that in the plan according to RTOG 0848.

**Table 3:** Dosimetric parameters of organ of risk for the proposed plan and RTOG 0848 plan in 4 simulated cases

	The proposed plan					RTOG 0848				
	Constraints	Case 1	Case 2	Case 3	Case 4	Constraints	Case 1	Case 2	Case 3	Case 4
left kidney						V18 < 65 %	34.12 %	46.95 %	47.47 %	42.25 %
right kidney						V18 < 50 %	42.27 %	42.85 %	37.06 %	35.36 %
Stomach	V15 < 9 cc	7.52 cc	4.41 cc	7.65 cc	0.00 cc	Dmax ≤ 58Gy	52.72Gy	52.40Gy	52.79Gy	34.55Gy
	V20 < 3 cc	1	0.26 cc	0.51 cc	0.00 cc	V56 < 10 %	0.00 %	0.00 %	0.00 %	0.00 %
						V52 < 15 %	0.04 %	0.07 %	0.05 %	0.00 %
Small intestine	V15 < 9 cc	8.27 cc	4.76 cc	3.24 cc	8.26 cc	Dmax ≤ 58Gy	55.67Gy	53.16Gy	53.26Gy	54.08Gy
	V20 < 3 cc	1.08 cc	1.82 cc	0.36 cc	1.12 cc	V56 < 10 %	0.00 %	0.00 %	0.00 %	0.00 %
						V52 < 15 %	8.83 %	3.42 %	0.25 %	2.87 %
Liver	V12 < 50 %	21.86 %	14.92 %	7.99 %	9.09 %	Dmean ≤ 30Gy	23.46Gy	16.57Gy	15.66Gy	15.86Gy
Spinal cord	V8 < 1 cc	0.04 cc	0.09 cc	0.01 cc	0.13 cc	V50 < 0.03 cc	0.00 cc	0.00 cc	0.00 cc	0.00 cc
Combined kidney	V12 < 75 %	48.27 %	37.87 %	36.89 %	49.68 %					
Large bowel	V15 < 9 cc	6.92 cc	3.22 cc	6.62	6.02 cc					
	V20 < 3 cc	1.60 cc	0.00 cc	0.00 cc	0.16 cc					
Proximal large bowel	V33 < 1 cc	0.00 cc	0.00 cc	0.00 cc	0.00 cc					
Proximal stomach	V33 < 1 cc	0.00 cc	0.00 cc	0.00 cc	0.00 cc					
Proximal small intestine	V33 < 1 cc	0.00 cc	0.00 cc	0.00 cc	0.00 cc					

*Abbreviations*: *RTOG* Radiation Therapy Oncology Group

**Table 4:** Target volume in the proposed plan and RTOG0848 plan

	PTV(cc)			PTV_modified(cc)		
	PTV(RTOG0848)	PTV-90	PTV-80	PTV_modified (RTOG0848)	PTV-90_modified	PTV-80_modified
Case 1	484.33	226.64	121.71	444.84	191.02	110.28
Case 2	368.54	190.49	103.62	356.26	166.83	94.66
Case 3	518.92	209.81	115.23	506.43	205.43	114.88
Case 4	586.39	251.9	140.61	556.43	224.61	130.95
Average	489.545	219.71	120.2925	465.99	196.9725	112.6925

*Abbreviations*: *PTV-90_modified* planning target volume covering 90 % plotted recurrences with avoidance of proximal organs at risk, *PTV-80_modified* planning target volume covering 80 % plotted recurrences with avoidance of proximal organs at risk

**Table 5:** Plan characteristics for the proposed plan and RTOG 0848 plan in 4 simulated cases

	Constraints	Case 1	Case 2	Case 3	Case 4
PTV-80_modified	V39.6 < 1 cc	0.72 cc	0.90 cc	0.87 cc	0.47 cc
	V33 ≥ 90 %	90 %	90 %	90 %	90 %
PTV-90_modified	V25 ≥ 90 %	98.64 %	98.75 %	93.33 %	98.81 %
PTV_modified (RTOG0848)	V50.4 ≥ 95 %	95 %	95 %	95 %	95 %

*Abbreviations*: *PTV-90_modified* planning target volume covering 90 % plotted recurrences with avoidance of proximal organs at risk, *PTV-80_modified* planning target volume covering 80 % plotted recurrences with avoidance of proximal organs at risk

**Table 6:** Comparison of dosimetric parameters between the proposed plan and RTOG 0848 plan in 4 simulated cases (cGy)

	The proposed plan			RTOG 0848			The proposed plan			RTOG 0848		
	D99	D50	D1	D99	D50	D1	D99	D50	D1	D99	D50	D1
	Case 1						Case 2					
PTV_modified (RTOG0848)				5010	5132	5303				5004	5101	5243
left kidney	251	1004	2234	1456	1797	3080	41	505	2182	138	1750	4625
right kidney	65	374	1601	578	1527	5299	40	392	2241	123	1477	4749
Stomach	92	676	1956	293	1898	4392	69	599	1654	225	2123	4865
Small intestine	66	699	1904	360	2801	5267	104	739	1614	382	3238	5147
Liver	29	526	3211	117	2477	5209	11	270	2737	75	1493	5137
Spinal cord	2	131	702	9	675	4564	2	72	697	1	275	3728
Large bowel	0	3	1420	0	9	5169	0	3	1246	0	22	2658
Proximal large bowel	74	628	1879	458	2166	5266	72	284	1739	260	1030	3845
Proximal stomach	104	688	1959	339	1915	4405	99	662	1671	389	2247	4850
Proximal small intestine	86	847	1937	643	3021	5276	104	739	1614	368	3025	5243
PTV-80_modified	2989	3571	3947				2978	3581	3934			
PTV-90_modified	2461	3362	3923				2457	3350	3904			
	Case 3						Case 4					
PTV_modified (RTOG0848)				5000	5132	5230				4995	5136	5249
left kidney	57	654	1629	271	1834	3618	90	836	1975	1312	1904	3821
right kidney	76	823	1904	404	1682	4443	68	631	1890	916	1717	4036
Stomach	19	128	1481	104	1108	5100	52	134	942	162	510	3197
Small intestine	37	500	1570	273	1874	5140	30	379	1537	364	1919	5206
Liver	19	222	1801	118	1576	5108	11	230	2116	103	1436	5172
Spinal cord	2	96	752	21	868	3624	1	67	738	4	485	3602
Large bowel	0	192	1500	0	1064	5009	0	5	1531	0	89	4366
Proximal large bowel	83	747	1582	566	2083	5052	62	583	1746	697	2400	4807
Proximal stomach	81	490	1600	733	2571	5112	110	394	1003	492	1699	3077
Proximal small intestine	70	793	1634	480	2235	5187	82	621	1716	591	2105	5257
PTV-80_modified	3110	3596	3949				2990	3557	3940			
PTV-90_modified	2380	3344	3924				2478	3350	3922			

*Abbreviations*: *D99* the dose 99 % of the volume received, *D50* the dose 50 % of the volume received, *D1* the dose 1 % of the volume received, *PTV-90_modified* planning target volume covering 90 % plotted recurrences with avoidance of proximal organs at risk, *PTV-80_modified* planning target volume covering 80 % plotted recurrences with avoidance of proximal organs at risk

## Discussion

So far there were limited studies focusing on recurrence pattern of pancreatic cancer. Herman et al. first demonstrated that a majority of local recurrences in postoperative patients with PHC are contained within a small region surrounding the CA and SMA and created an anatomic map with areas at the highest risk for local recurrence [13]. In their data, a majority of patients have undergone radiation. However, radiation therapy may reduce the risk of recurrence within the irradiation volume, thus influence the spatial location of local recurrence. Here, in this study, we followed the study of Herman et al. but only included patients without radiotherapy to rule out the impact of radiotherapy on the recurrence pattern.

In glioma, contouring the target volume according to the recurrence pattern has been proved feasible. Chang et al. have investigated the recurrence pattern in patients with glioblastoma after surgery and chemoradiotherapy. They successfully demonstrated that the smaller target volume would not increase the pattern of local failure [15, 16]. In pancreatic cancer, Heye et al. have reported the recurrence pattern in postoperative patients. They, from the viewpoint of radiologists, found the SMA is the leading structure for recurrence [17]. While in our study, a three-dimensional map of the local recurrences based on their relative coordinates to SMA or CA was further generated to guide target volume contouring for radiotherapy. Figure 1 suggests that the distribution of local recurrences between NA and CTA are similar in pancreatic cancer, and we found that the recurrence pattern have correlation with the location of primary tumor. The recurrence location of PTC seemed at the left of the CA and SMA and was far from them. It seems improper to create an adjuvant treatment volume for PTC based on expansions of the CA and SMA. We didn’t do further analysis of PBC as the patients number of PBC in our study is small. We then focused on the analysis of pancreatic head cancer. The target volumes were created by expanding the structure of the SMA and CA to cover 90 % and 80 % plotted recurrences (Fig. 2). As shown in Fig. 3, the target volume proposed in this study was much smaller than that in RTOG 0848. The low number of patients (n = 4 cases) used for the comparison to the RTOG protocol might be a limitation factor. However, the volume of PTV-80 was only one-fourth of that of RTOG 0848 PTV (Table 4), these cases exhibit good example. It is reasonable to apply these smaller treatment volumes in clinical practices because it might reduce radiation related toxicity and achieve dose escalation. The treatment plans according to this study and RTOG 0848 in 4 cases were further generated, which indicated the shrinked areas could potentially reduce the dose to OARs, then minimize the radiation related toxicity (Fig. 4, Tables 4 and 6).

To our knowledge, this study is the first research in pancreatic cancer which produced a map of local recurrences in postoperative patients who did not receive radiotherapy. It has been proved to be safe in rectal cancer to deliver 25Gy in 5-Gy fractions to the pelvic cavity which contains OARs such as small intestine and large bowel [18, 19]. In our study, PTV-90_modified was delivered a similar schedule as that in rectal cancer. PTV-80_modified was delivered higher dose (33 Gy in 6.6-Gy fractions) which was supported by clinical experience in other gastrointestinal malignancies. The BED of PTV-80_modified was 54.78Gy, which was similar to that of PTV (59.47 Gy) in RTOG 0848. OARs such as small intestine, the average dose 50 % of the volume received (D50) in the 4 cases was 5.79 Gy, while it was much higher in RTOG 0848 plan (24.73 Gy). Large bowel, stomach and other OARs also had lower average D50 than that in RTOG 0848 plan (Table 6). Therefore, with the proposed plan, higher dose to the target volume and lower radiation related toxicity to OARs is achievable.

Up to now, the areas already identified at risk of local recurrence in RTOG 0848 guidelines (aorta, portal vein, tumor bed, and pancreaticojejunostomy) are irradiated at standard doses in clinical practice. In this study, we demonstrated the feasibility of the smaller target volume for radiotherapy in patients with past-operative pancreatic head cancer. However, some limitations still existed. Firstly, recurrence was confirmed by follow-up CT or MRI, but not biopsy which was rarely used in clinical practice at this situation. Although we combined CA199, patients’ symptom, physical condition and Multidisciplinairy team for help, imaging diagnosis is still not golden standard. Secondly, the study was based on a static CT, the respiration- and peristalsis-induced shift in OARs was not taken into account. In daily clinical practice, 4D-CT should be utilized to eliminate this factor on dose distribution. Thirdly, we aim to from the viewpoint of evidence-based medicine to explore the target volume of adjuvant radiotherapy of pancreatic cancer, while the study size was not large enough. Fourthly, this study was retrospective analyses on historical cohort, which limited the conclusions. Therefore, there is a need to include more cases and to conduct larger-sample prospective studies on the pattern of recurrence of patients with pancreatic cancer after surgery in the future.

## Conclusions

We produce a map of local recurrence in postoperative patients with pancreatic head cancer. The areas at highest risk for local recurrence are much smaller than the standard adjuvant radiation target volumes based on RTOG consensus guidelines. This might provide reference for adjuvant radiotherapy in patients with pancreatic head cancer to achieve dose escalation and minimize radiation related toxicity.

## Abbreviations

CA: Celiac axis
CRT: Chemoradiation
CT: Abdominal Computed Tomography
CTA: Adjuvant chemotherapy alone
CTV: Clinical target volume
DRR: Digitally reconstructed radiograph
MRI: Magnetic Resonance Imaging
NA: No adjuvant therapy
NCCR: National Central Cancer Registry
OARs: Organs at risk
OIs: Regions of interest
PBC: Pancreatic body cance
PCA: Pancreatic cancer
PHC: Pancreatic head cancer
PJ: Pancreaticojejunostomy
PTC: Pancreatic tail cancer
PTV: Planning target volume
PV: Portal vein
RTOG: Radiation Therapy Oncology Group
SAHZU: The Second Affiliated Hospital, Zhejiang University School of Medicine
SBRT: Stereotactic body radiation therapy
SMA: Superior mesenteric artery
